# Redetermined structure, inter­molecular inter­actions and absolute configuration of royleanone

**DOI:** 10.1107/S1600536811011457

**Published:** 2011-04-07

**Authors:** Hoong-Kun Fun, Suchada Chantrapromma, Abdul Wahab Salae, Ibrahim Abdul Razak, Chatchanok Karalai

**Affiliations:** a X-ray Crystallography Unit, School of Physics, Universiti Sains Malaysia, 11800 USM, Penang, Malaysia; b Crystal Materials Research Unit, Department of Chemistry, Faculty of Science, Prince of Songkla University, Hat-Yai, Songkhla 90112, Thailand

## Abstract

The structure of the title diterpenoid, C_20_H_28_O_3_, {systematic name: (4b*S*,8a*S*)-3-hy­droxy-2-isopropyl-4b,8,8-trimethyl-4b,5,6,7,8,8a,9,10-octa­hydro­phenanthrene-1,4-dione} is confirmed [Eugster *et al.* (1993 ▶). Private communication (refcode HACGUN). CCDC, Union Road, Cambridge] and its packing is now described. Its absolute structure was established by refinement against data collected with Cu radiation: the two stereogenic centres both have *S* configurations. One cyclo­hexane ring adopts a chair conformation whereas the other cyclo­hexane ring is in a half-chair conformation and the benzoquinone ring is slightly twisted. An intra­molecular O—H⋯O hydrogen bond generates an *S*(5) ring motif. In the crystal, mol­ecules are linked into chains along [010] by O—H⋯O hydrogen bonds and weak C—H⋯O inter­actions. The packing also features C⋯O [3.131 (3) Å] short contacts.

## Related literature

For the previous determination of the title structure, see: Eugster *et al.* (1993 ▶). For ring conformations, see: Cremer & Pople (1975 ▶). For bond-length data, see: Allen *et al.* (1987 ▶). For background to *Verbenaceae* plants and the bioactivity of diterpenoids, see: Bunluepuech & Tewtrakul (2009 ▶); Edwards *et al.* (1962 ▶); Kabouche *et al.* (2007 ▶); Suresh *et al.* (2011 ▶); Slamenová *et al.* (2004 ▶); Tezuka *et al.* (1998 ▶). For a related structure, see: Razak *et al.* (2010 ▶). For hydrogen-bond motifs, see: Bernstein *et al.* (1995) ▶. For the stability of the temperature controller used in the data collection, see Cosier & Glazer, (1986 ▶).
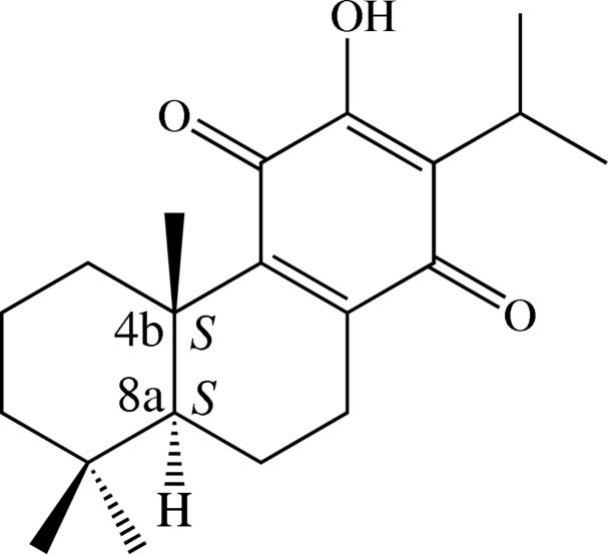


## Experimental

### 

#### Crystal data

C_20_H_28_O_3_
*M* *_r_* = 316.42
Monoclinic, 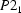

*a* = 10.2247 (2) Å
*b* = 7.6353 (1) Å
*c* = 10.7292 (2) Å
β = 97.992 (1)°
*V* = 829.48 (2) Å^3^
*Z* = 2
Cu *K*α radiation
μ = 0.66 mm^−1^
*T* = 100 K
0.52 × 0.31 × 0.15 mm

#### Data collection

Bruker APEX DUO CCD diffractometer
Absorption correction: multi-scan (*SADABS*; Bruker, 2009 ▶) *T*_min_ = 0.726, *T*_max_ = 0.909
5901 measured reflections
2390 independent reflections
2375 reflections with *I* > 2σ(*I*)
*R*_int_ = 0.021

#### Refinement

*R*[*F* ^2^ > 2σ(*F* ^2^)] = 0.036
*wR*(*F* ^2^) = 0.098
*S* = 1.06
2390 reflections
217 parameters
1 restraint
H atoms treated by a mixture of independent and constrained refinement
Δρ_max_ = 0.35 e Å^−3^
Δρ_min_ = −0.20 e Å^−3^
Absolute structure: Flack (1983 ▶) 699 Friedel pairs
Flack parameter: 0.11 (19)

### 

Data collection: *APEX2* (Bruker, 2009) ▶; cell refinement: *SAINT* (Bruker, 2009) ▶; data reduction: *SAINT* ▶; program(s) used to solve structure: *SHELXTL* (Sheldrick, 2008 ▶); program(s) used to refine structure: *SHELXTL*; molecular graphics: *SHELXTL*; software used to prepare material for publication: *SHELXTL* (Sheldrick, 2008 ▶) and *PLATON* (Spek, 2009 ▶).

## Supplementary Material

Crystal structure: contains datablocks global, I. DOI: 10.1107/S1600536811011457/hb5812sup1.cif

Structure factors: contains datablocks I. DOI: 10.1107/S1600536811011457/hb5812Isup2.hkl

Additional supplementary materials:  crystallographic information; 3D view; checkCIF report

## Figures and Tables

**Table 1:** Hydrogen-bond geometry (Å, °)

*D*—H⋯*A*	*D*—H	H⋯*A*	*D*⋯*A*	*D*—H⋯*A*
O2—H1*O*2⋯O1	0.88 (4)	2.05 (3)	2.5977 (18)	119 (3)
O2—H1*O*2⋯O3^i^	0.88 (4)	2.35 (4)	3.1079 (19)	145 (3)
C1—H1*A*⋯O1	0.97	2.38	2.993 (2)	120
C7—H7*A*⋯O1^ii^	0.97	2.51	3.131 (3)	122
C17—H17*B*⋯O2	0.96	2.49	3.071 (2)	119
C20—H20*A*⋯O1	0.96	2.47	3.125 (2)	125

Symmetry codes: (i) 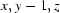; (ii) 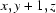.

**Fig. 1.**
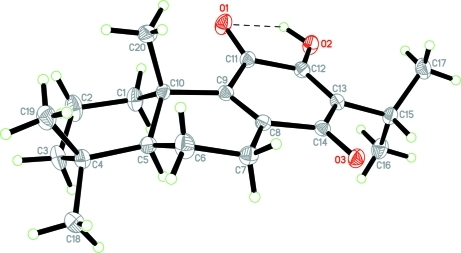
The structure of (I), showing 40% probability displacement ellipsoids. The hydrogen bond is shown as a dashed line.

**Fig. 2.**
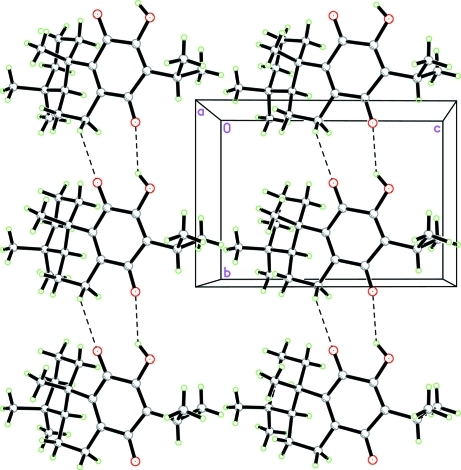
The crystal packing of (I) viewed along the a axis, showing [010] chains. Hydrogen bonds are shown as dashed lines.

## supplementary crystallographic information

### Comment

The extracts of Verbenaceae plants have been found to possess anti-HIV-1 integrase activity (Bunluepuech & Tewtrakul, 2009) and cytotoxicity (Suresh *et al.*, 2011). During the course of our study of chemical constituents and bioactive compounds from *Premna obtusifolia* (Verbenaceae), the title diterpenoid (I), which is known as royleanone (Edwards *et al.*, 1962; Tezuka *et al.*, 1998) was isolated from the roots of this plant. Compound (I) was reported to show significant biological properties such as antioxidant (Kabouche *et al.*, 2007) and cytotoxic activities (Slame˘nová *et al.*, 2004). The absolute configuration of (I) was determined by making use of the anomalous scattering of Cu Kα*X*-radiation with the Flack parameter being refined to 0.11 (19). We herein report the crystal structure of (I).

The molecule of (I) has three fused six membered rings (Fig. 1). The two cyclohexane rings are *trans* fused. One cyclohexane ring (C1–C5/C10) is in a standard chair conformation whereas the other (C5–C10) is in half chair conformation, with the C5 and C6 atoms having the deviation of 0.396 (2) and -0.323 (2) Å, respectively from the plane through C7–C10 atoms and the puckering parameters Q = 0.563 (2) Å, θ = 55.20 (19)° and φ = 16.1 (2)° (Cremer & Pople, 1975). The benzoquinone ring (C8–C9/C11–C14/O1/O3) is slightly twisted with the maximum deviations of -0.091 (1) and 0.055 (2) Å for atoms C9 and C13, respectively, and with the puckering parameters Q = 0.1474 (19) Å, θ = 72.9 (7)° and φ = 86.8 (8)° (Cremer & Pople, 1975). The O1, O2 and O3 atoms lie close to the mean plane of the C8–C9/C11–C14 ring with the *r.m.s.* of 0.0870 (1) Å. The bond angles around C8, C9, C12 and C13 are indicative of *sp*^2^ hybridization for these atoms. The orientation of the propanyl group is described by the torsion angles C12–C13–C15–C16 = -69.8 (2) and C12–C13–C15–C17 = 54.2 (2) °. Intramolecular O2—H1O2···O1 hydrogen bond (Table 1) generate S(5) ring motif (Fig. 1) (Bernstein *et al.*, 1995). The bond distances and angles in (I) are within normal ranges (Allen *et al.*, 1987) and comparable with a related structure (Razak *et al.*, 2010). The absolute configuration at atoms C1 and C5 or positions 4b and 8a of royleanone are both *S*, which agrees with the previous stereochemistry of royleanone (Kabouche *et al.*, 2007; Slame˘nová *et al.*, 2004; Tezuka *et al.*, 1998). The S,S configurations are also consistent with those in a related structure (Razak *et al.*, 2010).

In the crystal of (I) (Fig. 2), the molecules are linked into chains along the [0 1 0] through O2—H1O2···O3 hydrogen bond and C7—H7A···O1 weak interaction (Fig. 2 and Table 1). The crystal is stabilized by these interactions together with C···O[3.131 (3) Å] short contacts.

### Experimental

The air-dried roots of *premna obtusifolia* (4.5 kg) were extracted with hexane (2 × 20 l) at room temperature. The combined extracts were concentrated under reduced pressure to give a dark yellow extract (40.0 g) which was subjected to quick column chromatography (QCC) over silica gel using solvents of increasing polarity from n-hexane to EtOAc to afford 7 fractions (F1-F7). Fraction F2 was further purified by quick column chromatography using hexane, yielding the title compound (6.1 mg). Yellow blocks were recrystallized from CH_2_Cl_2_ by the slow evaporation of the solvent at room temperature after several days, M.p 451-453 K.

### Refinement

The hydroxy H atom was located from the difference map and refined isotropically. The remaining H atoms were placed in calculated positions with (C—H) = 0.98 for CH, 0.97 for CH_2_ and 0.96 Å for CH_3_ atoms. The *U*_iso_ values were constrained to be 1.5*U*_eq_ of the carrier atom for methyl H atoms and 1.2*U*_eq_ for the remaining H atoms. A rotating group model was used for the methyl groups. The highest residual electron density peak is located at 0.74 Å from C10 and the deepest hole is located at 0.72 Å from C2. 699 Friedel pairs were used to determine the absolute configuration.

### Crystal data

**Table:**

C_20_H_28_O_3_	*F*(000) = 344
*M**_r_* = 316.42	*D*_x_ = 1.267 Mg m^−^^3^
Monoclinic, *P*2_1_	Melting point = 451–453 K
Hall symbol: P 2yb	Cu *K*α radiation, λ = 1.54178 Å
*a* = 10.2247 (2) Å	Cell parameters from 2390 reflections
*b* = 7.6353 (1) Å	θ = 5.6–72.1°
*c* = 10.7292 (2) Å	µ = 0.66 mm^−^^1^
β = 97.992 (1)°	*T* = 100 K
*V* = 829.48 (2) Å^3^	Block, yellow
*Z* = 2	0.52 × 0.31 × 0.15 mm

### Data collection

**Table:**

Bruker APEX Duo CCD diffractometer	2390 independent reflections
Radiation source: sealed tube	2375 reflections with *I* > 2σ(*I*)
graphite	*R*_int_ = 0.021
φ and ω scans	θ_max_ = 72.1°, θ_min_ = 5.6°
Absorption correction: multi-scan (*SADABS*; Bruker, 2009)	*h* = −12→12
*T*_min_ = 0.726, *T*_max_ = 0.909	*k* = −9→6
5901 measured reflections	*l* = −13→12

### Refinement

**Table:**

Refinement on *F*^2^	Secondary atom site location: difference Fourier map
Least-squares matrix: full	Hydrogen site location: inferred from neighbouring sites
*R*[*F*^2^ > 2σ(*F*^2^)] = 0.036	H atoms treated by a mixture of independent and constrained refinement
*wR*(*F*^2^) = 0.098	*w* = 1/[σ^2^(*F*_o_^2^) + (0.0639*P*)^2^ + 0.2006*P*] where *P* = (*F*_o_^2^ + 2*F*_c_^2^)/3
*S* = 1.06	(Δ/σ)_max_ = 0.001
2390 reflections	Δρ_max_ = 0.35 e Å^−^^3^
217 parameters	Δρ_min_ = −0.20 e Å^−^^3^
1 restraint	Absolute structure: Flack (1983) 699 Friedel pairs
Primary atom site location: structure-invariant direct methods	Flack parameter: 0.11 (19)

### Special details

**Table:**

Experimental. The crystal was placed in the cold stream of an Oxford Cryosystems Cobra open-flow nitrogen cryostat (Cosier & Glazer, 1986) operating at 100.0 (1) K.
Geometry. All esds (except the esd in the dihedral angle between two l.s. planes) are estimated using the full covariance matrix. The cell esds are taken into account individually in the estimation of esds in distances, angles and torsion angles; correlations between esds in cell parameters are only used when they are defined by crystal symmetry. An approximate (isotropic) treatment of cell esds is used for estimating esds involving l.s. planes.
Refinement. Refinement of F^2^ against ALL reflections. The weighted R-factor wR and goodness of fit S are based on F^2^, conventional R-factors R are based on F, with F set to zero for negative F^2^. The threshold expression of F^2^ > 2sigma(F^2^) is used only for calculating R-factors(gt) etc. and is not relevant to the choice of reflections for refinement. R-factors based on F^2^ are statistically about twice as large as those based on F, and R- factors based on ALL data will be even larger.

### Fractional atomic coordinates and isotropic or equivalent isotropic displacement parameters (Å^2^)

**Table:**

	*x*	*y*	*z*	*U*_iso_*/*U*_eq_	
O1	0.64538 (13)	−0.0889 (2)	0.48735 (12)	0.0254 (3)	
O2	0.54777 (12)	−0.05136 (18)	0.25211 (12)	0.0219 (3)	
H1O2	0.567 (3)	−0.139 (5)	0.304 (3)	0.055 (9)*	
O3	0.59784 (11)	0.55637 (18)	0.31995 (11)	0.0202 (3)	
C1	0.91254 (18)	0.0149 (3)	0.61639 (17)	0.0244 (4)	
H1A	0.8633	−0.0932	0.6003	0.029*	
H1B	0.9513	0.0438	0.5414	0.029*	
C2	1.02342 (19)	−0.0129 (3)	0.72689 (19)	0.0293 (4)	
H2A	0.9853	−0.0529	0.7997	0.035*	
H2B	1.0828	−0.1033	0.7048	0.035*	
C3	1.10107 (17)	0.1532 (3)	0.76032 (17)	0.0258 (5)	
H3A	1.1496	0.1824	0.6916	0.031*	
H3B	1.1652	0.1310	0.8341	0.031*	
C4	1.01699 (15)	0.3115 (3)	0.78684 (15)	0.0194 (4)	
C5	0.90137 (15)	0.3285 (3)	0.67650 (14)	0.0181 (4)	
H5A	0.9442	0.3520	0.6020	0.022*	
C6	0.80962 (18)	0.4855 (3)	0.68588 (18)	0.0264 (4)	
H6A	0.7458	0.4577	0.7419	0.032*	
H6B	0.8607	0.5859	0.7199	0.032*	
C7	0.73866 (16)	0.5283 (3)	0.55608 (16)	0.0194 (4)	
H7A	0.6623	0.6004	0.5646	0.023*	
H7B	0.7972	0.5959	0.5108	0.023*	
C8	0.69435 (15)	0.3689 (2)	0.48092 (15)	0.0155 (4)	
C9	0.72317 (14)	0.2047 (2)	0.52206 (14)	0.0153 (4)	
C10	0.81709 (15)	0.1624 (2)	0.64288 (14)	0.0153 (3)	
C11	0.65384 (15)	0.0604 (3)	0.44783 (15)	0.0174 (4)	
C12	0.58810 (16)	0.0954 (3)	0.31623 (15)	0.0175 (4)	
C13	0.57151 (15)	0.2572 (3)	0.26820 (15)	0.0165 (4)	
C14	0.61740 (15)	0.4041 (3)	0.35330 (15)	0.0156 (3)	
C15	0.51364 (15)	0.3012 (3)	0.13372 (14)	0.0184 (4)	
H15A	0.5037	0.4287	0.1280	0.022*	
C16	0.61027 (18)	0.2461 (3)	0.04345 (16)	0.0260 (4)	
H16A	0.6936	0.3034	0.0671	0.039*	
H16B	0.5750	0.2790	−0.0409	0.039*	
H16C	0.6226	0.1215	0.0477	0.039*	
C17	0.37759 (17)	0.2199 (3)	0.09225 (18)	0.0262 (4)	
H17A	0.3187	0.2518	0.1508	0.039*	
H17B	0.3856	0.0947	0.0900	0.039*	
H17C	0.3431	0.2621	0.0099	0.039*	
C18	1.10451 (18)	0.4757 (3)	0.78830 (19)	0.0294 (5)	
H18A	1.1833	0.4592	0.8472	0.044*	
H18B	1.0570	0.5755	0.8129	0.044*	
H18C	1.1279	0.4948	0.7058	0.044*	
C19	0.97379 (18)	0.2988 (3)	0.91827 (15)	0.0266 (4)	
H19A	1.0493	0.3138	0.9813	0.040*	
H19B	0.9350	0.1860	0.9281	0.040*	
H19C	0.9101	0.3886	0.9274	0.040*	
C20	0.73179 (17)	0.1029 (3)	0.74287 (16)	0.0265 (4)	
H20A	0.6730	0.0115	0.7087	0.040*	
H20B	0.6813	0.2003	0.7666	0.040*	
H20C	0.7880	0.0598	0.8155	0.040*	

### Atomic displacement parameters (Å^2^)

**Table:**

	*U*^11^	*U*^22^	*U*^33^	*U*^12^	*U*^13^	*U*^23^
O1	0.0347 (7)	0.0149 (7)	0.0236 (6)	−0.0044 (6)	−0.0064 (5)	0.0030 (5)
O2	0.0283 (6)	0.0142 (8)	0.0208 (6)	−0.0021 (5)	−0.0050 (5)	−0.0011 (5)
O3	0.0217 (6)	0.0152 (7)	0.0221 (6)	−0.0003 (5)	−0.0024 (4)	0.0026 (5)
C1	0.0284 (8)	0.0194 (11)	0.0228 (8)	0.0053 (8)	−0.0062 (7)	−0.0049 (7)
C2	0.0307 (9)	0.0224 (11)	0.0313 (10)	0.0092 (9)	−0.0082 (7)	−0.0048 (9)
C3	0.0194 (8)	0.0331 (13)	0.0227 (8)	0.0065 (8)	−0.0046 (6)	−0.0044 (8)
C4	0.0183 (7)	0.0220 (11)	0.0169 (7)	−0.0007 (8)	−0.0006 (6)	−0.0001 (7)
C5	0.0189 (7)	0.0198 (10)	0.0152 (7)	−0.0025 (7)	0.0009 (6)	0.0001 (7)
C6	0.0310 (9)	0.0199 (10)	0.0258 (9)	0.0003 (8)	−0.0055 (7)	−0.0054 (8)
C7	0.0224 (7)	0.0148 (10)	0.0207 (8)	−0.0005 (7)	0.0016 (6)	−0.0011 (7)
C8	0.0143 (7)	0.0157 (10)	0.0163 (7)	−0.0009 (6)	0.0020 (6)	−0.0002 (6)
C9	0.0147 (6)	0.0167 (10)	0.0145 (7)	−0.0008 (6)	0.0020 (5)	−0.0005 (6)
C10	0.0172 (7)	0.0158 (9)	0.0124 (7)	−0.0013 (7)	0.0005 (6)	0.0006 (6)
C11	0.0177 (7)	0.0158 (10)	0.0182 (7)	0.0007 (7)	0.0015 (6)	0.0008 (7)
C12	0.0176 (7)	0.0167 (11)	0.0178 (8)	−0.0011 (7)	0.0016 (6)	−0.0020 (7)
C13	0.0141 (7)	0.0179 (10)	0.0170 (8)	0.0000 (6)	0.0004 (6)	−0.0003 (7)
C14	0.0132 (6)	0.0157 (9)	0.0181 (7)	0.0007 (6)	0.0029 (6)	−0.0001 (7)
C15	0.0220 (7)	0.0160 (10)	0.0161 (7)	0.0003 (7)	−0.0016 (6)	0.0009 (7)
C16	0.0301 (9)	0.0294 (12)	0.0181 (8)	0.0049 (8)	0.0020 (6)	0.0026 (7)
C17	0.0224 (8)	0.0256 (11)	0.0277 (8)	0.0000 (8)	−0.0065 (6)	−0.0001 (8)
C18	0.0268 (9)	0.0307 (12)	0.0281 (9)	−0.0091 (9)	−0.0056 (7)	0.0027 (9)
C19	0.0277 (8)	0.0341 (12)	0.0167 (8)	−0.0016 (8)	−0.0013 (6)	−0.0050 (8)
C20	0.0225 (8)	0.0388 (13)	0.0181 (8)	−0.0087 (8)	0.0020 (6)	0.0034 (8)

### Geometric parameters (Å, °)

**Table:**

O1—C11	1.224 (2)	C8—C14	1.506 (2)
O2—C12	1.349 (2)	C9—C11	1.481 (2)
O2—H1O2	0.88 (4)	C9—C10	1.536 (2)
O3—C14	1.225 (2)	C10—C20	1.542 (2)
C1—C2	1.537 (2)	C11—C12	1.501 (2)
C1—C10	1.542 (2)	C12—C13	1.341 (3)
C1—H1A	0.9700	C13—C14	1.481 (3)
C1—H1B	0.9700	C13—C15	1.519 (2)
C2—C3	1.513 (3)	C15—C17	1.532 (2)
C2—H2A	0.9700	C15—C16	1.535 (2)
C2—H2B	0.9700	C15—H15A	0.9800
C3—C4	1.533 (3)	C16—H16A	0.9600
C3—H3A	0.9700	C16—H16B	0.9600
C3—H3B	0.9700	C16—H16C	0.9600
C4—C19	1.538 (2)	C17—H17A	0.9600
C4—C18	1.539 (3)	C17—H17B	0.9600
C4—C5	1.558 (2)	C17—H17C	0.9600
C5—C6	1.534 (3)	C18—H18A	0.9600
C5—C10	1.548 (3)	C18—H18B	0.9600
C5—H5A	0.9800	C18—H18C	0.9600
C6—C7	1.514 (2)	C19—H19A	0.9600
C6—H6A	0.9700	C19—H19B	0.9600
C6—H6B	0.9700	C19—H19C	0.9600
C7—C8	1.495 (2)	C20—H20A	0.9600
C7—H7A	0.9700	C20—H20B	0.9600
C7—H7B	0.9700	C20—H20C	0.9600
C8—C9	1.348 (3)		
			
C12—O2—H1O2	107 (2)	C9—C10—C5	106.69 (14)
C2—C1—C10	112.12 (15)	C20—C10—C5	115.45 (14)
C2—C1—H1A	109.2	C1—C10—C5	107.17 (13)
C10—C1—H1A	109.2	O1—C11—C9	123.94 (15)
C2—C1—H1B	109.2	O1—C11—C12	116.59 (16)
C10—C1—H1B	109.2	C9—C11—C12	119.46 (16)
H1A—C1—H1B	107.9	C13—C12—O2	123.80 (15)
C3—C2—C1	111.93 (18)	C13—C12—C11	122.80 (16)
C3—C2—H2A	109.2	O2—C12—C11	113.40 (16)
C1—C2—H2A	109.2	C12—C13—C14	116.67 (14)
C3—C2—H2B	109.2	C12—C13—C15	125.48 (17)
C1—C2—H2B	109.2	C14—C13—C15	117.82 (16)
H2A—C2—H2B	107.9	O3—C14—C13	120.92 (15)
C2—C3—C4	114.57 (15)	O3—C14—C8	118.62 (15)
C2—C3—H3A	108.6	C13—C14—C8	120.41 (16)
C4—C3—H3A	108.6	C13—C15—C17	113.86 (15)
C2—C3—H3B	108.6	C13—C15—C16	109.82 (14)
C4—C3—H3B	108.6	C17—C15—C16	110.11 (15)
H3A—C3—H3B	107.6	C13—C15—H15A	107.6
C3—C4—C19	111.12 (16)	C17—C15—H15A	107.6
C3—C4—C18	107.69 (15)	C16—C15—H15A	107.6
C19—C4—C18	106.43 (16)	C15—C16—H16A	109.5
C3—C4—C5	108.09 (14)	C15—C16—H16B	109.5
C19—C4—C5	114.68 (13)	H16A—C16—H16B	109.5
C18—C4—C5	108.58 (15)	C15—C16—H16C	109.5
C6—C5—C10	109.26 (13)	H16A—C16—H16C	109.5
C6—C5—C4	114.94 (15)	H16B—C16—H16C	109.5
C10—C5—C4	116.62 (16)	C15—C17—H17A	109.5
C6—C5—H5A	104.9	C15—C17—H17B	109.5
C10—C5—H5A	104.9	H17A—C17—H17B	109.5
C4—C5—H5A	104.9	C15—C17—H17C	109.5
C7—C6—C5	109.12 (15)	H17A—C17—H17C	109.5
C7—C6—H6A	109.9	H17B—C17—H17C	109.5
C5—C6—H6A	109.9	C4—C18—H18A	109.5
C7—C6—H6B	109.9	C4—C18—H18B	109.5
C5—C6—H6B	109.9	H18A—C18—H18B	109.5
H6A—C6—H6B	108.3	C4—C18—H18C	109.5
C8—C7—C6	113.02 (17)	H18A—C18—H18C	109.5
C8—C7—H7A	109.0	H18B—C18—H18C	109.5
C6—C7—H7A	109.0	C4—C19—H19A	109.5
C8—C7—H7B	109.0	C4—C19—H19B	109.5
C6—C7—H7B	109.0	H19A—C19—H19B	109.5
H7A—C7—H7B	107.8	C4—C19—H19C	109.5
C9—C8—C7	122.99 (14)	H19A—C19—H19C	109.5
C9—C8—C14	121.79 (15)	H19B—C19—H19C	109.5
C7—C8—C14	115.20 (15)	C10—C20—H20A	109.5
C8—C9—C11	116.69 (14)	C10—C20—H20B	109.5
C8—C9—C10	123.65 (15)	H20A—C20—H20B	109.5
C11—C9—C10	119.60 (16)	C10—C20—H20C	109.5
C9—C10—C20	107.53 (12)	H20A—C20—H20C	109.5
C9—C10—C1	109.58 (13)	H20B—C20—H20C	109.5
C20—C10—C1	110.27 (16)		
			
C10—C1—C2—C3	−56.7 (2)	C4—C5—C10—C9	−172.64 (13)
C1—C2—C3—C4	54.2 (2)	C6—C5—C10—C20	−64.48 (19)
C2—C3—C4—C19	76.8 (2)	C4—C5—C10—C20	67.96 (19)
C2—C3—C4—C18	−167.00 (15)	C6—C5—C10—C1	172.24 (13)
C2—C3—C4—C5	−49.9 (2)	C4—C5—C10—C1	−55.33 (17)
C3—C4—C5—C6	−177.96 (16)	C8—C9—C11—O1	−162.38 (16)
C19—C4—C5—C6	57.5 (2)	C10—C9—C11—O1	14.7 (2)
C18—C4—C5—C6	−61.40 (19)	C8—C9—C11—C12	17.2 (2)
C3—C4—C5—C10	52.25 (18)	C10—C9—C11—C12	−165.74 (13)
C19—C4—C5—C10	−72.3 (2)	O1—C11—C12—C13	169.20 (16)
C18—C4—C5—C10	168.81 (14)	C9—C11—C12—C13	−10.4 (2)
C10—C5—C6—C7	−68.58 (19)	O1—C11—C12—O2	−10.7 (2)
C4—C5—C6—C7	158.12 (15)	C9—C11—C12—O2	169.71 (14)
C5—C6—C7—C8	40.90 (19)	O2—C12—C13—C14	177.82 (14)
C6—C7—C8—C9	−4.3 (2)	C11—C12—C13—C14	−2.1 (2)
C6—C7—C8—C14	177.17 (14)	O2—C12—C13—C15	−4.1 (2)
C7—C8—C9—C11	169.78 (13)	C11—C12—C13—C15	176.03 (14)
C14—C8—C9—C11	−11.8 (2)	C12—C13—C14—O3	−174.92 (15)
C7—C8—C9—C10	−7.2 (2)	C15—C13—C14—O3	6.8 (2)
C14—C8—C9—C10	171.21 (13)	C12—C13—C14—C8	7.7 (2)
C8—C9—C10—C20	105.79 (19)	C15—C13—C14—C8	−170.58 (13)
C11—C9—C10—C20	−71.10 (19)	C9—C8—C14—O3	−177.84 (15)
C8—C9—C10—C1	−134.34 (17)	C7—C8—C14—O3	0.68 (19)
C11—C9—C10—C1	48.77 (19)	C9—C8—C14—C13	−0.4 (2)
C8—C9—C10—C5	−18.63 (19)	C7—C8—C14—C13	178.12 (13)
C11—C9—C10—C5	164.48 (13)	C12—C13—C15—C17	54.2 (2)
C2—C1—C10—C9	170.62 (16)	C14—C13—C15—C17	−127.68 (17)
C2—C1—C10—C20	−71.2 (2)	C12—C13—C15—C16	−69.8 (2)
C2—C1—C10—C5	55.2 (2)	C14—C13—C15—C16	108.33 (18)
C6—C5—C10—C9	54.93 (16)		

### Hydrogen-bond geometry (Å, °)

**Table:**

*D*—H···*A*	*D*—H	H···*A*	*D*···*A*	*D*—H···*A*
O2—H1O2···O1	0.88 (4)	2.05 (3)	2.5977 (18)	119 (3)
O2—H1O2···O3^i^	0.88 (4)	2.35 (4)	3.1079 (19)	145 (3)
C1—H1A···O1	0.97	2.38	2.993 (2)	120
C7—H7A···O1^ii^	0.97	2.51	3.131 (3)	122
C17—H17B···O2	0.96	2.49	3.071 (2)	119
C20—H20A···O1	0.96	2.47	3.125 (2)	125

Symmetry codes:  (i) *x*, *y*−1, *z*; (ii) *x*, *y*+1, *z*.

## References

[bb1] Allen, F. H., Kennard, O., Watson, D. G., Brammer, L., Orpen, A. G. & Taylor, R. (1987). *J. Chem. Soc. Perkin Trans. 2*, pp. S1–19.

[bb16] Bernstein, J., Davis, R. E., Shimoni, L. & Chang, N.-L. (1995). *Angew. Chem. Int. Ed. Engl.* **34**, 1555–1573.

[bb2] Bruker (2009). *APEX2*, *SAINT* and *SADABS* Bruker AXS Inc., Madison, Wisconsin, USA.

[bb3] Bunluepuech, K. & Tewtrakul, S. (2009). *Songklanakarin J. Sci. Technol* **31**, 289–292

[bb4] Cosier, J. & Glazer, A. M. (1986). *J. Appl. Cryst.* **19**, 105–107.

[bb5] Cremer, D. & Pople, J. A. (1975). *J. Am. Chem. Soc.* **97**, 1354–1358.

[bb6] Edwards, O. E., Feniak, G. & Los, M. (1962). *Can. J. Chem* **40**, 1540–1546.

[bb7] Eugster, C. H., Ruedi, P., Tanudjaja, T., Bieri, J. H., Prewo, R. & Linden, A. (1993). Private communication (refcode HACGUN). CCDC, Union Road, Cambridge.

[bb8] Flack, H. D. (1983). *Acta Cryst.* A**39**, 876–881.

[bb9] Kabouche, A., Kabouche, Z., Öztürk, M., Kolak, U. & Topçu, G. (2007). *Food Chem* **102**, 1281–1287.

[bb10] Razak, I. A., Salae, A. W., Chantrapromma, S., Karalai, C. & Fun, H.-K. (2010). *Acta Cryst.* E**66**, o1566–o1567. 10.1107/S1600536810020544 PMC3006754 21587809

[bb11] Sheldrick, G. M. (2008). *Acta Cryst.* A**64**, 112–122. 10.1107/S0108767307043930 18156677

[bb12] Slamenová, D., Masterová, I., Lábaj, J., Horváthová, E., Kubala, P., Jakubíková, J. & Wsólová, L. (2004). *Basic Clin. Pharmacol. Toxicol* **94**, 282–290. 10.1111/j.1742-7843.2004.pto940605.x 15228500

[bb13] Spek, A. L. (2009). *Acta Cryst.* D**65**, 148–155. 10.1107/S090744490804362X PMC2631630 19171970

[bb14] Suresh, G., Babu, K. S., Rao, V. R. S., Rao, M. S. A., Nayak, V. L. & Ramakrishna, S. (2011). *Tetrahedron. Lett.* **52**, 1273–1276.

[bb15] Tezuka, Y., Kasimu, R., Li, J. X., Basnet, P., Tanaka, K., Namba, T. & Kadota, S. (1998). *Chem. Pharm. Bull* **46**, 107–112.

